# Application of contrast-enhanced ultrasound in renal space-occupying lesion puncture biopsy

**DOI:** 10.1186/s12880-023-01137-9

**Published:** 2023-11-08

**Authors:** Zhi-Ying Jia, Feng Yang, Wei Zhang, Wei-Wei Li

**Affiliations:** 1 Department of Ultrasound, The Affiliated Tumor Hospital of Xinjiang Medical University, No. 789 of Suzhou East Street, Xinshi District, 830011 Urumqi, China; 2 Urology Surgery, The Affiliated Tumor Hospital of Xinjiang Medical University, No. 789 of Suzhou East Street, Xinshi District, 830011 Urumqi, China

**Keywords:** Biopsy, Contrast-enhanced ultrasound, Puncture, Renal tumors, Ultrasound

## Abstract

**Objective:**

The purpose of this study was to investigate the utility of contrast-enhanced ultrasound (CEUS) in percutaneous renal space-occupying lesion puncture biopsy.

**Methods:**

Ultrasound (US)-guided percutaneous needle biopsies were performed on 55 patients with renal space-occupying lesions, and the results were analyzed retrospectively. The US group included 36 patients receiving conventional US, and the contrast-enhanced ultrasound (CEUS) group included 22 patients, including 19 patients receiving CEUS directly and 3 patients receiving additional enhanced ultrasound due to the first conventional ultrasound puncture failure. The relevant data were subjected to statistical analysis.

**Results:**

The results of this study showed that the successful rate of obtaining effective tissue (100% vs. 75%) and the puncture accuracy (100% vs. 88.89%) in CEUS group were significantly higher than those in US group (*P* < 0.05). CEUS-guided puncture biopsy of renal mass, especially in the case of urothelial carcinoma of the renal pelvis, outperforms conventional ultrasound, and the difference was statistically significant (*P* < 0.05).

**Conclusion:**

Percutaneous renal space-occupying lesion puncture biopsies aided by CEUS yield more effective tissue and improved puncture accuracy.

## Introduction

The rapid advancement of interventional ultrasound has led to its widespread application in the diagnosis and treatment of clinical diseases in recent years. Needle biopsies of abdominal, pelvic, and superficial lesions are routinely performed using ultrasound guidance due to the numerous benefits of the technique, including its ease of use, adaptability, lack of radiation, and high precision. In order to obtain a definitive diagnosis and set the stage for subsequent treatment, the percutaneous renal space-occupying lesion puncture biopsy has been widely implemented in clinical practice [1]. However, accurate pathological results at the first puncture can be difficult to achieve due to the high risk of bleeding from the rich renal blood supply [2]. The extent of tumor necrosis and vascularization can be reflected by contrast-enhanced ultrasound [3]. Therefore, to investigate the potential clinical utility of contrast-enhanced ultrasound (CEUS) in enhancing the precision of puncture biopsy for renal space-occupying lesions, we retrospectively compared the results of 22 cases in which CEUS was used to guide the biopsy with those of 36 cases in which conventional ultrasound (US) was used to guide the biopsy.

## Data and methods

### General data

We selected 55 patients hospitalized in the urinary surgery department of the Affiliated Tumor Hospital of Xinjiang Medical University from March 2021 to September 2022 who had not been previously diagnosed with renal space-occupying lesions or had received any related treatment. After admission, 55 patients underwent imaging studies (CT, CTA, MRI, US, etc.) to evaluate the presence of any renal space-occupying lesions, and subsequent evaluation included a US-guided or CEUS-guided renal space-occupying lesion puncture biopsy. The 55 patients included 35 men and 20 women, with ages ranging from 36 to 84 years with an average of (62.0 ± 11.3) years. There were 31 right kidney cases and 24 left kidney cases, both of which involved unilateral renal masses. Renal space-occupying lesions ranged in size from 2.0 to 19.9 cm in diameter, with an average diameter of (7.73 ± 3.21) cm. Pathological diagnosis was obtained through puncturing the specimen, and patients were enrolled into the US group and the CEUS group in a sequential fashion based on the selected ultrasound-guided technique. The US group included 36 patients receiving conventional US, and the contrast-enhanced ultrasound (CEUS) group included 22 patients, including 19 patients receiving CEUS directly and 3 patients receiving additional enhanced ultrasound due to the first conventional ultrasound puncture failure.

### Instruments and methods

The color Doppler ultrasound diagnostic equipment models were GE LOGIQ E9 and SAMSUNG XR80A, with each operating with a convex array probe operating at frequencies between 1 and 6 MHz and 1 to7 MHz, respectively. GE LOGIQ E9 was equipped with ultrasound contrast function and time-intensity curve (TIC) analysis software.

The patients were typically positioned in the lateral decubitus or prone position using a soft pillow to raise their waist. In the US group, the affected kidney was comprehensively scanned by conventional ultrasound, and the relationship between the location, size, shape, edge, internal echo, internal and peripheral blood flow distribution and course, adjacent tissue structure, renal hilar blood vessels, and renal space-occupying lesions was observed. A safe path was selected, and the body was appropriately positioned, and the skin puncture point was marked on the body. After conventional ultrasound, the patients in the CEUS group underwent CEUS-guided renal space-occupying lesion puncture biopsy without changing the position. SonoVue produced by Bracco Company, Italy, was used as the ultrasonic contrast agent. The SonoVue contrast agent was mixed with 5.0 ml normal saline and oscillated until a suspension of sulfur hexafluoride microbubbles formed. A 5 ml syringe was used to extract 1.5 ml suspension, which was administered to the patient through the median cubital vein, and then 5 ml normal saline was administered to flush the syringe tube. The perfusion of renal space-occupying lesions contrast agent was dynamically observed in real time, and segmented into perfusion and non-perfusion areas based on enhancement. Based on the CEUS manifestations, the perfusion area was used as the puncture target, a safe path was chosen, the non-enhancement area was avoided, and the position was adjusted before the body of the patient was positioned.

Ultrasound-guided puncture biopsy of renal space-occupying lesions: After US or CEUS, the skin of the patient was disinfected at the site of the draping without the patient changing the position, and after local anesthesia with 2% lidocaine, three to four tissue samples were collected using the BARD automatic biopsy gun and 18G (model 1820) disposable biopsy needle. The tissues were sent to the pathology lab in a formalin-fixed solution. After confirming that the patient was safe to return to the ward, routine scanning was performed to check for bleeding around the kidney, and the patient was then dressed in sterile gauze fixed by pressure at the puncture point and sent back to the ward for 6–8 h. The collected puncture tissue was considered satisfactory if there were sufficient tissues that could be effectively viewed under a pathological microscope, and a clear pathological diagnosis could be obtained.

All patients underwent various examinations before surgery and signed the informed consent form for needle biopsy. Patients undergoing CEUS also signed an informed consent form for CEUS. The puncture biopsy was completed by urologists and ultrasound doctors.

### Statistical method

SPSS24.0 statistical software was used for statistical analysis of relevant data. Measurement data that conform to the normal distribution are expressed as mean ± standard deviation; the comparison between groups was made by T test, and the counting data was tested using the chi-squared test and nonparametric test. The confidence level was set at α = 0.05, and P < 0.05 was considered as statistically significant.

## Results

**Fig. 1:**
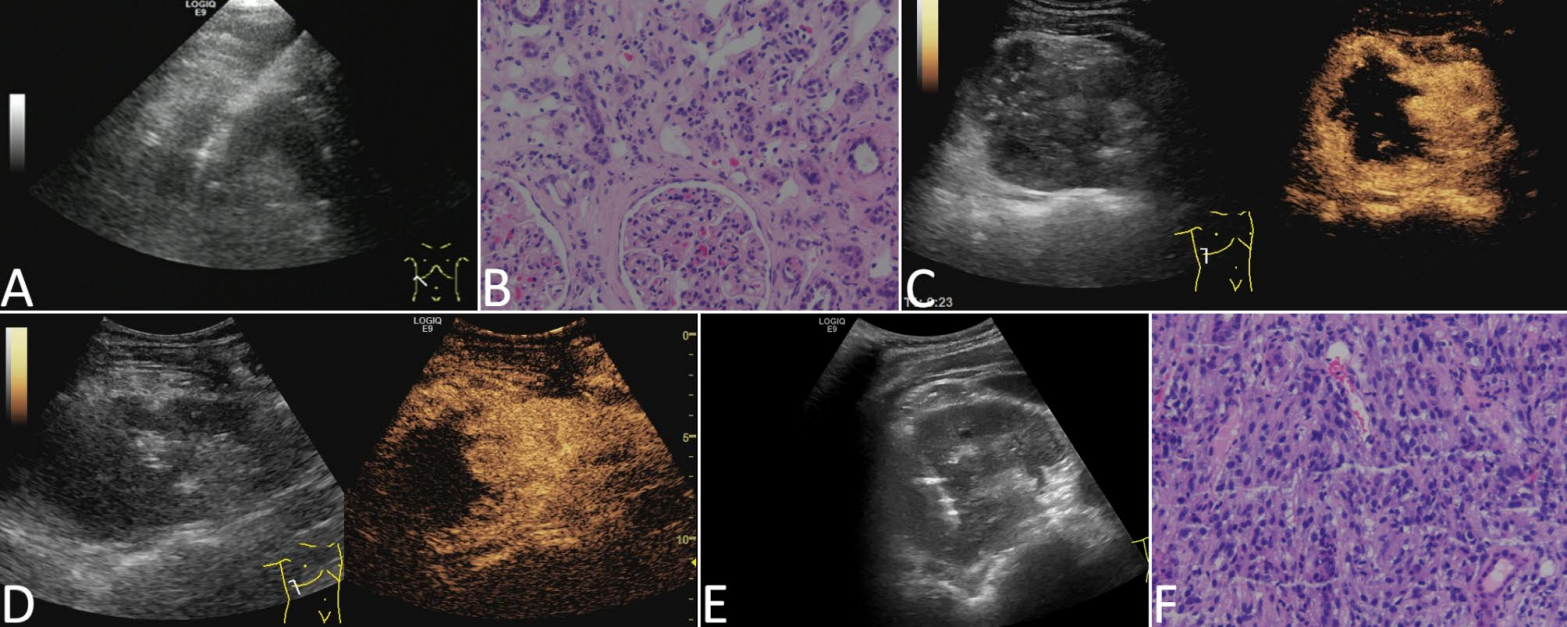
1 remedial case achieved by CEUS-guided puncture. The case was a patient who had a space-occupying lesion in the upper pole of the right kidney, about 9.5 × 7.8 cm in size, with irregular shape and unclear boundary. The liquefaction area was not found by grayscale ultrasound. (**a**) The right kidney biopsy guided by conventional ultrasound was performed for the first time. (**b**) The effective tissue components of the first puncture specimen were few and no tumor components were found under the pathological microscope (HE staining, ×200 times). (**c**-**d**) CEUS in the second biopsy showed large necrosis in the lesion, and the puncture target was located in the area with contrast agent perfusion. (**e**) The puncture biopsy of the enhanced area in the lesion was carried out immediately. (**f**) Under the pathological microscope, diffuse infiltration of tumor cells stained with cytoplasm (HE staining, ×200 times) could be seen

Among the 36 conventional US-guided needle biopsies, 32 cases were diagnosed pathologically. We failed to obtain samples in 4 cases; among them, we successfully obtained the samples in 3 cases after they underwent CEUS-guided needle biopsy (see Fig. 1), and we obtained the sample successfully in the remaining case through CT-guided needle biopsy.

Histopathological results were successfully obtained in 19 cases of CEUS-guided needle biopsy. Based on the adopted ultrasound-guided method, the cases in this study were divided into two groups, namely, 36 cases in the US group and 22 cases in the CEUS group (Among them, there were 3 cases in which conventional US-guided puncture biopsy failed for the first time and CEUS-guided puncture biopsy was performed successfully the second time).

The data in Table 1 were statistically analyzed and independent sample T test was performed for the measurement data and chi-squared test for the enumeration data.

**Table 1:** Comparison of general data between the two groups of cases

General data	Ultrasonic method		t/*χ*^2^	*P*
	US(*n* = 36)	CEUS(*n* = 22)		
Age (years)	61.31 ± 11.19	62.86 ± 11.49	0.509	0.613
Sex (cases)				
Male	21	16	1.410	0.235
Female	15	6		
Location (cases)				
Left kidney	15	9	0.021	0.885
Right kidney	21	13		
Maximum diameter (cm)	7.60 ± 3.39	7.93 ± 2.98	0.370	0.706
Grouping by maximum diameter (cases)				
≤ 4 cm	4	1		0.640
> 4 cm	32	21		

Fisher’s exact probability method was adopted for the maximum diameter grouping data table as the expected count of two cells was less than 5

There was no statistically significant difference in age, gender, lesion location, and lesion size between the two groups of cases (*P* > 0.05). The results of non-parametric Wilcoxon rank sum test for the two groups of non-normal distribution count data displayed in Table 2 demonstrate that there was no statistically significant difference between the pathological results in the two groups of cases.

**Table 2:** Nonparametric test of histopathological results of the two groups of cases

Pathological diagnosis	Ultrasonic method		Z	*P*
	US (n = 36)	CEUS (n = 22)		
Clear-cell carcinoma	20	12	-1.227	0.220
Chromophobe cell carcinoma	1	0		
Renal cell carcinoma	5	3		
Urothelial carcinoma	2	6		
Metastatic carcinoma	2	0		
Inflammatory lesion	1	0		
Malignant tumour	1	1		
No tumor cells were found	4	0		

Common clear cell carcinomas were the dominant ones. Further, 49 cases which were identified as renal cell carcinoma and urothelial carcinoma in the two groups were extracted and subjected to inter-group chi-squared test, and the difference was statistically significant (*P* = 0.045) (P < 0.05). The proportion of urothelial carcinoma in the CEUS group was higher than that in the US group, which suggests that CEUS is more suitable as a guiding method for puncture biopsy in the case of urothelial carcinoma. As shown in Table 3, the puncture tissue specimens of 27 cases in the US group were found to be satisfactory for diagnosis, and the accuracy rate of routine histopathological diagnosis was 75%.

**Table 3:** Comparison of puncture result between the two groups of cases

Puncture result	Ultrasonic method		*P*
	US (n = 36)	CEUS (n = 22)	
Puncture success	27 (75.0%)	22 (100%)	0.037
Puncture failure	4 (11.1%)	0	
Small amount of tissue specimens	5 (13.9%)	0	

Fisher’s exact probability method was adopted as the expected count of four cells was less than 5

The effectively available tissues of 5 punctured specimens were small. The final diagnosis was obtained by further improving immunohistochemistry, and the puncture accuracy was improved to 88.89%. The CEUS-guided needle biopsies (including 3 cases of failure of US-guided needle biopsy) were obtained with a histopathological diagnosis accuracy of 100%. The statistical results also showed that the difference in the biopsy guidance accuracy for renal space-occupying lesions guided between the two methods was statistically significant (*P* < 0.05). The CEUS group was better than the US group, and it was easier to obtain satisfactory histological results.

## Discussion

The diagnosis of kidney disease is the most common application of the percutaneous renal biopsy, which has been practiced in China for decades and whose technology is now mature enough to provide an important basis for clinical diagnosis and treatment [4]. In recent years, as the concept of treating renal tumors has evolved, needle biopsies have become increasingly common as a means of clarifying the nature of the disease prior to treatment, particularly for patients who are too far along in the disease process to undergo surgery. This can guide the clinical development of corresponding treatment strategies and the use of targeted drugs [5, 6].

Puncture biopsy of renal space-occupying lesions is most commonly performed using ultrasound guidance because of its ease of use, adaptability, and real-time dynamics. However, conventional ultrasonic gray scale and color Doppler flow have a low accuracy in judging the necrotic or liquefied area of the tumor, which is often mistaken for the solid part of the tumor. Though multi-point sampling is accomplished by varying the puncture angle, ineffective tissues or sampling failure can still occur, forcing a second biopsy at the patient’s expense and exposing them to a higher degree of risk during the second puncture. Tumor perfusion distribution can be displayed directly with CEUS, [7, 8] and the area perfused with the contrast agent can be taken as the puncture target, thereby effectively avoiding necrotic or liquefied parts and improving the quality of puncture specimens. Although prior research has shown that CEUS can aid in increasing puncture biopsy accuracy, [9, 10] rarely has CEUS-guided puncture biopsy of renal space-occupying lesions been reported. Histopathological results were obtained in one puncture for all cases in the CEUS group, and for the three cases in which US-guided puncture had failed, a second puncture using CEUS had the desired effect.

Based on our findings, gender, age, and tumor location have no bearing on puncture success. The majority of patients in our study had metastatic renal cancer or were in the advanced stage of the disease. The average maximum diameter of the lesions was (7.73 ± 3.21) cm, and the volume was relatively large. Small tumors accounted for only 5 of the cases. It is commonly held that smaller tumors are more difficult to accurately puncture than larger ones. However, this becomes a problem when the volume of the tumor is too large [11]—Large lesions are more likely to be complicated with bleeding and necrosis, which may explain why the accuracy of puncture biopsy decreases when the diameter is greater than 5.0 or 6.0 cm.

Consistent with the findings of this paper, renal cell carcinoma is the most common renal malignancy, accounting for over 90% of adult renal malignancies, and clear cell carcinoma is the most common subtype, accounting for 70–80% of renal cell carcinoma [12]. Renal pelvic urothelial carcinoma is a rare form of cancer that begins in the urothelium of the renal pelvis. The renal pelvis and calyx system is notoriously difficult to diagnose accurately with conventional ultrasound. Although there was no statistically significant difference between the US and CEUS groups in terms of overall pathological results, there was a significant difference in the composition ratio of urothelial carcinoma (2 cases in the US group and 6 cases in the CEUS group). In the US group, two of the four failed biopsies after the second puncture were confirmed to be urothelial carcinoma (50%). The reasons for the failure of the first puncture were considered in the retrospective analysis of cases as follows: ① It was challenging to determine the extension of the lesion using only gray-scale ultrasound, [13] leading to the operator misjudging the puncture direction, but CEUS made up for the shortcomings of conventional ultrasound by clearly displaying the lesion’s location in the renal pelvis and partial invasive growth, as well as its boundary with the renal parenchyma; ② The puncture failed due to the high risk and low success rate of percutaneous intrapelvic tumor puncture. Most of the samples were taken at the periphery of the lesion because the operating doctors were inexperienced and worried about complications. Based on the pathological findings, it was the tissue surrounding the tumor that prevented a successful puncture.

In the US group, 5 cases did not obtain sufficient available tissue by routine ultrasound-guided puncture, and pathological diagnosis was achieved by further immunohistochemistry. In contrast, all patients obtained sufficient available tissue by puncture in the CEUS group, indicating better sampling capacity under CEUS puncture. The reason for this may be that, CEUS is better than conventional ultrasound at judging the active part of the puncture target—the solid lesion with rich blood vessels. According to a study conducted by Chen et al. on 52 isolated kidney specimens, the puncture accuracy could be improved by using more needles, with a success rate of sampling reaching 100% when using three needles [14]. Coaxial biopsy needles minimize the risk of puncture because they only require a single insertion and allow for repeated material retrieval along the same needle track [15]. If the tissues samples are unsatisfactory under naked eye observation, the number of puncture needles can be appropriately increased to four to five, to get a larger amount of usable tissue. According to some scholars, the selection of the puncture needle type also affects the success rate of the puncture [16]. In this study, we used an 18-G biopsy needle in both groups, so the choice of needle size cannot account for the observed differences in outcomes.

This study has the following limitations: First, the number of included cases is small, and most of the tumors present are in advanced stage; Second, the volume of renal space-occupying lesions contained in the specimens was large. Therefore, it is necessary to further explore the application of contrast-enhanced ultrasound in puncture biopsy of kidney space-occupying lesions ≤ 4 cm in larger sample size studies.

## Conclusion

In summary, CEUS can clearly show the tumor outline and internal necrotic liquefaction area, ensuring high puncture accuracy and guiding further clinical treatment. Especially for renal pelvic space occupying lesions, CEUS-guided percutaneous puncture biopsy of renal space occupying lesions is helpful to obtain more effective tissue specimens.

## Data Availability

The datasets used and/or analysed during the current study available from the corresponding author on reasonable request. We declared that materials described in the manuscript, including all relevant raw data, will be freely available to any scientist wishing to use them for non-commercial purposes, without breaching participant confidentiality.
